# RNA-seq analysis of blood from cave- and surface-dwelling *Astyanax* morphs reveal diverse transcriptomic responses to normoxic rearing

**DOI:** 10.3389/fphys.2025.1617136

**Published:** 2025-07-17

**Authors:** Tyler E. Boggs, Lydia R. Bucher, Joshua B. Gross

**Affiliations:** Department of Biological Sciences, University of Cincinnati, Cincinnati, OH, United States

**Keywords:** hypoxia, subterranean, GO terms, enrichment analysis, normoxia

## Abstract

Adaptive responses to hypoxia are likely accompanied by highly diverse changes in gene expression. Here, we examined the transcriptomic regulation in blood samples derived from independently-derived captive cave-dwelling fish. These fish are members of the species *Astyanax mexicanus*, which comprises two morphs: an obligate subterranean form, and a “surface-dwelling” form that lives in rivers and streams located near cave localities. These morphs diverged ∼20,000–200,000 years ago, and cavefish derived from multiple, distinct cave localities have adapted to life in hypoxic waters. Here, we focused on captive-reared *Astyanax* morphs since elevated hemoglobin levels persist in cavefish despite rearing in the normoxic conditions of a laboratory. A GO enrichment analysis revealed several instances of convergent gene regulation between some, but not all, cavefish populations. This finding suggests that different gene expression patterns have evolved in response to hypoxia across geologically-distinct cave localities. Additionally, we identified differential regulation of numerous genes of the canonical hypoxic response pathway. Interestingly, some genes activating this pathway were expressed lower in captive-reared cavefish. These patterns of gene expression may have evolved in cavefish as a consequence of negative pleiotropic consequences associated with prolonged *hif* gene expression. At present, it is unknown whether this finding is a function of captivity, or whether these expression patterns are also present in wild populations. Collectively, this work provides new insights to the transcriptomic regulation of hypoxia tolerance using a cavefish model evolving in distinct oxygenated environments.

## Introduction

A number of transcriptomic studies in teleosts reveal that adaptation to low oxygen is accompanied by diverse changes in gene regulation. These gene expression alterations impact diverse processes such as metabolic suppression, intracardiac cooperation, increase in gill surface area, vasculature growth, and red blood cell overproduction. Hemoglobin family members are common targets of hypoxic stress, including the preferential expression of *hemoglobin* (*hb*) isoforms with unusually high oxygen affinity and sensitivity to allosteric regulators [reviewed in Nikinmaa and Rees (2005), Xiao (2015), Fago and Jensen (2015)]. Many of these traits are controlled by changes in expression of the hypoxia inducible factor (*Hif*) (Mandic et al., 2021).

**FIGURE 1:**
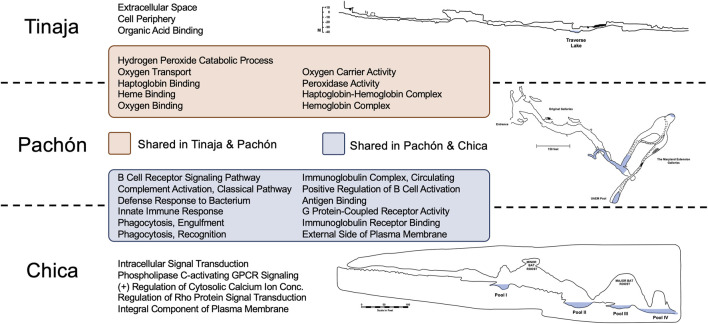
GO Enrichment revealed many shared and unique terms across cave populations relating to oxygen transport and immune function. Statistically overrepresented terms in Tinaja and Pachón cavefish compared to surface fish largely represented oxygen binding and transport. The majority of overrepresented terms in Pachón and Chica cavefish related to immune function. Interestingly, there were no overlapping terms between Tinaja and Chica compared to surface fish. The distinct geography and environmental histories of these caves have likely impacted global gene expression patterns differently in *Astyanax* cavefish.

Hypoxia is present in a variety of environments including frozen ponds, reef platforms at low tide, high altitude, deep-sea, aquatic environments with algal blooms, and caves (Storz, 2018). Here, we examined adaptation to hypoxia in cavefish with ancestors that evolved in a limestone cave complex in the Sierra de El Abra region of northeastern Mexico (Figure 1). Over 30 caves populated by cavefish populations are found in this region (Miranda-Gamboa et al., 2023). EL Abra caves are characterized by limited or absent light, minimal nutrition, and lower dissolved oxygen compared to the terrestrial environment. Each cave, however, is unique with respect to formation process, elevation, size, inhabitant fauna, volume of terrestrial input, and other factors (Elliott, 2018). Despite these differences, cavefish derived from these habitats evolve a number of convergent phenotypic features.

These fish are members of the species *Astyanax mexicanus*, which comprises two morphotypes: an obligate cave-dwelling fish lacking eyes and pigmentation, and a terrestrial “surface” fish with stereotypical teleost features. It is estimated that ∼20,000–200,000 years ago these cave environments were colonized by surface-dwelling lineages (Herman et al., 2018; Fumey et al., 2018; Moran et al., 2023; Garduño-Sánchez et al., 2023). Extant cave and surface morphs inhabit starkly contrasting environments, providing the opportunity to examine evolutionary changes in closely-related morphotypes inhabiting environments marked by different levels of oxygen.

van der Weele and Jeffery (2022) discovered juvenile cavefish from the Pachón cave locality grow normally in hypoxic conditions, but surface fish do not. By 36 h post fertilization (hpf), cavefish produce more red blood cells than surface fish. This red blood cell expansion is accompanied by increased expression of certain *hemoglobin* genes, expanded hematopoietic domains, and elevated expression of several *hif* gene family members. Interestingly, prior work has shown that adult *Astyanax* cavefish, reared in captivity, show increased hemoglobin protein concentration in three different populations (Pachón, Tinaja, and Chica) compared to surface fish (Boggs et al., 2022). This concentration of adult hemoglobin is underpinned, in part, by larger red blood cells. However, elevated hemoglobin protein levels are mediated by the expression of different *hemoglobin* gene family members (Boggs and Gross, 2025).

Here, we examined how hypoxic adaptation impacts gene regulation by measuring broad scale transcriptomic regulation. We focused our attention to captive-reared *Astyanax* morphs since hemoglobin elevation persists in cave morphs, despite being reared in normoxic conditions. The results of a gene enrichment analysis of the blood transcriptomes of different morphs revealed shared enrichment patterns between Pachón and Chica cavefish, and Pachón and Tinaja cavefish (however Chica and Tinaja cavefish showed no overlap). Notably, many instances of overlap likely reflect convergent increases in expression of hemoglobin genes. However, we also identified numerous genes associated with canonical hypoxia response pathways. Many genes normally activating these pathways were expressed lower in cavefish compared to surface fish, and certain genes typically suppressing these pathways were expressed higher in cavefish. These surprising patterns may reflect the negative consequences that can arise as a function of prolonged *hif* expression. At present, it is unclear if these findings are a function of the captive conditions in which cavefish are reared in the lab, and whether these observations translate to natural populations as well. In any respect, this work provides news insight to the transcriptomic architecture of hypoxia tolerance, through use of a unique model that permits intraspecific comparison of morphs evolving in different oxygenated environments.

## Results and discussion

### Convergent and divergent regulation suggests cavefish suppress canonical hypoxia response pathways in normoxic captivity

We examined transcriptional gene regulation of blood by performing statistical overrepresentation analyses of Gene Ontology (GO) terms using PANTHERdb (Mi et al., 2019; Thomas et al., 2022). Accordingly, we scored annotated genes demonstrating significant two-fold (or higher) differences in gene expression in each cave population relative to surface fish. This resulted in six analyses, i.e., three pair-wise comparisons performed for both over- and under-expression. GO terms enrichments (FDR adjusted p-value <0.05) from each analysis were compared to identify convergent/divergent expression patterns between cave populations. A prior study using the same dataset provided expression validation through analysis of five genes subjected to quantitative real-time PCR (qPCR) and calculated delta Cq using *ssr3* as the reference gene [see Boggs and Gross (2025)]. This study revealed an average correlation coefficient of 0.89, indicating a strong relationship (Cohen et al., 2009) and validation of our RNA-seq dataset.

Interestingly, we discovered substantial overlap between Tinaja and Pachón, and Pachón and Chica (Figure 1). These overlapping sets included every identified GO term for Pachón, however no overlap was observed for Chica and Tinaja cavefish. Many GO terms shared between Tinaja and Pachón cavefish were significant due to *hemoglobin* genes [see Boggs and Gross (2025)] including: oxygen transport, heme binding, oxygen binding, and hemoglobin complex (Figure 1). Overlapping terms between Pachón and Chica mostly reflected terms associated with immune system function, including: defense response to bacterium, innate immune response, phagocytosis recognition, and antigen binding.

These results were not entirely surprising given that prior GO enrichment studies in *Astyanax* identified convergent mechanisms of cave adaptation, including broad development processes (Riddle et al., 2020), metabolism (Krishnan et al., 2020), and immunity (Krishnan et al., 2022). Additionally, the number and diversity of genes within a test list affects the outcome of overrepresentation studies (Wijesooriya et al., 2022). Given that whole blood is a highly complex tissue (capable of predicting an estimated 60% of gene expression for dozens of tissues) (Basu et al., 2021), this likely impacted the statistical outcomes of the analysis.

We further aimed to investigate genes of potential interest that may not have been detected in these GO analyses. Accordingly, we created four lists representing genes that are biologically-relevant to hypoxia including: genes expressed higher or lower in all examined cave populations compared to surface fish (Table 1) and genes expressed higher and lower in Tinaja and Pachón compared to surface fish (Table 2), while excluding Chica, given their similarity in hemoglobin expression. A literature search for each of these genes was conducted to provide any potential relevance to adaptation to hypoxic caves.

**TABLE 1:** Genes of interest shared in Chica, Tinaja, and Pachón and divergent from Surface fish.

Higher in cavefish			Lower in cavefish		
Ensembl ID	Gene name	Relevance	Ensembl ID	Gene name	Relevance
ENSAMXG00000025285	*tcimb*	Enhances NF-kB activity. Regulates hematopoietic stem cells. Knockouts had smaller but more numerous erythrocytes. (Jung et al., 2014)	ENSAMXG00000035038	*iscu*	Suppression in normoxia caused a shift to glycolysis and enhanced cell survival. (Favaro et al., 2010)
ENSAMXG00000030775	*tnnt2b*	Upregulated during hypoxia and putatively prevents excessive angiogenesis. (Watson et al., 2013)	ENSAMXG00000043108	*mt2_2*	Contributes to nitric oxide signaling and is overexpressed during hypoxia. (Yamasaki et al., 2007)
ENSAMXG00000020270	*lonrf3*	Contains a RING finger domain. Identified in QTL and GWAS studies as a candidate for hypoxia tolerance. (San et al., 2021; Prchal et al., 2023)	ENSAMXG00000011699	*ctsba*	HIF-1a binds to ctsba promotor and drives expression. (Xiaofei et al., 2018)
ENSAMXG00000035358	*tp53inp1*	HIF-1a activity is reduced by p53. (Zhou et al., 2015)	ENSAMXG00000021444	*lgmn*	Induced during hypoxia. Depletion led to reduced cell proliferation and increased apoptosis. (Clees et al., 2022)
ENSAMXG00000036037	*tcf20*	Expression closely linked to HIF-3a, an inhibitor of HIF-1a and HIF-2a. (Yang et al., 2015; Diao et al., 2022)	ENSAMXG00000035776	*b3gnt2a*	Downregulated in hypoxic carotid arteries. Known to influence cell proliferation. (Goyal and Longo, 2014)
ENSAMXG00000042715	*ddit4*	Inhibits mTOR pathways. (Fingar et al., 2002; Foltyn et al., 2019)	ENSAMXG00000043965	*aqp7*	Reduced expression increases apoptosis and myocardial infarct size. (Ishihama et al., 2021)
ENSAMXG00000019906	*cemip*	Expression is increased during hypoxia leading to enhanced cell migration. (Evensen et al., 2015)	ENSAMXG00000020315	*rragca*	Contributes to the activation of mTOR. (Chun and Kim, 2021)
ENSAMXG00000008364	*hbae*	Embryonic *hemoglobin* - oxygen transporter. (Storz, 2016)	ENSAMXG00000018717	*mef2d*	Transcription factor involved in hypoxic signaling in the cardiovascular system and nitric oxide signaling in neurons. (Estrella et al., 2015)
ENSAMXG00000041047	*rnh1*	Blocks ANG (angiogenin) signaling. ANG is normally upregulated during hypoxia. (Kishimoto et al., 2012; Sheng and Xu, 2016)	ENSAMXG00000034098	*akr1b1.1*	Inhibition resulted in decreased cell migration specific to hypoxia. (Tammali et al., 2011; Khayami et al., 2020)
ENSAMXG00000006257	*bbox1*	Knockdowns induced a deficiency of an mTOR pathway. (Arsham et al., 2003; Brugarolas et al., 2004)	ENSAMXG00000013404	mlf1*	Influences HSC differentiation. Overexpression interrupts development and differentiation of erythrocytes. (Winteringham et al., 2004; Li et al., 2023)

**TABLE 2:** Genes of interest shared in Tinaja and Pachón and divergent from Surface fish.

Higher in cavefish			Lower in cavefish		
Ensembl ID	Gene name	Relevance	Ensembl ID	Gene name	Relevance
ENSAMXG00000029151	*hbaa*	Adult hemoglobin - oxygen transporters	ENSAMXG00000043907	*hbe1_4*	Embryonic hemoglobin - oxygen transporter
ENSAMXG00000029181	*hbaa2_2*		ENSAMXG00000039076	*rhag*	CO2 channel on erythrocytes. CO2 binds HbA, decreasing affinity for O2, aiding delivery of O2 to tissues
ENSAMXG00000037475	*hbaa2_1*		ENSAMXG00000033903	*lamp2*	Chaperone that mediates HIF-1a. Decreased expression lowers HIF-1a abundance
ENSAMXG00000034763	*hbba2*		ENSAMXG00000037819	*pcbp2*	Depletion leads to accumulation of HIF1 transcription factors owing to impaired degradation mechanisms
ENSAMXG00000029578	*hbe1_2*	Embryonic hemoglobin - oxygen transporter	ENSAMXG00000007272	*hif1al2*	Encodes Hypoxia Inducible Factor subunit alpha, the most well characterized hypoxia response protein
ENSAMXG00000032394	*rgcc*	Induced by hypoxia. Contributes to differentiation of VEGF and FGF pathways resulting in anti-angiogenesis	ENSAMXG00000002219	*sf3b1*	Facilitates binding of HIF to hypoxia response elements to activate target gene expression
ENSAMXG00000010569	*a2m*	LncRNA regulates *IL1R2* to lessen hypoxic injury in cardiomyocytes	ENSAMXG00000001895	*eif5*	Transcription factor essential for the activation of HIF-1a in hypoxia
ENSAMXG00000002726	*atf5b*	Transcription factor upregulated during hypoxia that serves as a regulator of neuroprogenitor cell proliferation	ENSAMXG00000039259	*pora*	Putatively regulates EPO through HIF activation as well as VEGF during hypoxia
ENSAMXG00000030111	*mlphb*	Known to be involved in the HIF pathway and revealed as a candidate gene for high altitude adaptation in gelada monkeys	ENSAMXG00000019342	*hif1ab*	Encodes Hypoxia Inducible Factor subunit alpha, the most well characterized hypoxia response protein
ENSAMXG00000042466	*selenow1*	Deficiency of selenium can induce HIF and NF-kB pathways. Selenoproteins mediate the biological effects of selenium	ENSAMXG00000010550	mlf2*	Putatively functions similarly to *mlf1*, influencing HSC differentiation

Consistent with prior findings (Boggs and Gross, 2025), many *hemoglobin* genes were expressed higher in cavefish compared to surface fish (Tables 1 and 2) with the vast majority expressed higher only in Tinaja and Pachón (relative to Chica and Surface). We also identified numerous genes associated with canonical hypoxia response pathways. Interestingly, many genes normally activating these pathways were expressed lower in cavefish compared to surface fish, and genes typically suppressing these pathways were expressed higher in cavefish (Tables 1, 2). Notably, two *hypoxia inducible factor* (*hif)* genes (*hif1al2* and *hif1ab*) and multiple genes contributing to HIF signaling, including *cathepsin Ba* (*ctsba), lysosomal associated membrane protein 2 (lamp2), splicing factor 3b subunit 1 (sf3b1), eukaryotic translation initiation factor 5 (eif5), and p450 (cytochrome) oxidoreductase a (pora)* were expressed lower in cavefish compared to surface fish. Additionally, two genes known to suppress HIF signaling (*tp53inp1* and *tcf20*) were expressed higher in cavefish compared to surface fish.

In normoxic conditions, *hif* is continuously transcribed, but is controlled post-translationally by prolyl hydroxylase (PHD) and von Hippel-Lindau (VHL) proteins. During hypoxia, PHD activity is inhibited and Hif is not degraded. Thus, *hif* transcript abundance is not necessarily representative of Hif activity in mammals (Semenza and Wang, 1992; Maxwell et al., 1999; Ivan et al., 2001; Jaakkola et al., 2001; Bruick and McKnight, 2001; Epstein et al., 2001). In the Chinese sucker (*Myxocyprinus asiaticus*), a study revealed increased *hif* transcription is required to prevent degradation of Hif during hypoxia (Chen et al., 2012). Having said this, a recent study uncovered diverse reports of *hifa* mRNA abundance in fish exposed to hypoxia, as a likely function of varying methodologies (Murphy and Rees, 2024). Nevertheless, elasmobranch fish conditioned to hypoxia express *hif* higher than individuals that have not experienced hypoxia (Rytkönen et al., 2012). Additionally, certain *hif* family members are expressed higher in Pachón cavefish embryos (after normoxic rearing or exposure to hypoxia) than in surface fish (van der Weele and Jeffery, 2022). Thus, we were initially surprised to find that adult cavefish express two *hif* family members much lower than surface fish and express other known hypoxia response genes in similar, counterintuitive, patterns. In light of varying reports of *hifa* transcription in fish (Murphy and Reese, 2024), it will be essential to better characterize protein levels of hif1a in forthcoming studies through the use of Western blot analyses.

One explanation for these observed patterns may be the negative consequences associated with prolonged expression of *hif*. *Hif* is linked to many human pathologies including tumorigenesis, cardiovascular, metabolic, and reproductive diseases [reviewed in Chen et al. (2020)]. In mice, pharmacological knock-down of Hif protein relieved symptoms of rheumatoid arthritis (Hu et al., 2020). Hif pathways can also impair major histocompatibility complex function in culture, leading to an inability to recognize and eliminate cancerous and other harmful cells (Sethumadhavan et al., 2017). Additionally, Hif proteins influence ion fluctuations and homeostasis in fish, a well-characterized mechanism to conserve energy during hypoxia [reviewed in Pelster and Egg (2018)]. Thus, future work in *Astyanax* may determine if downregulation of *hif* and other known hypoxia response pathways are advantageous in cavefish to save energy, maintain proper immune function, and prevent disease and inflammation.

Prior work in *Astyanax* revealed that oxygen levels are a good deal lower in cave waters compared to surface waters (Boggs and Gross, 2025). An important consideration for this study is the fact that all experimental animals were reared in normoxic conditions. Indeed, our putative Chica cavefish were acquired from a commercial vendor, and therefore it is not possible for us to determine the extent to which transcriptomic changes are a function of assimilation to captivity. Interestingly, a number of cave populations maintain significantly elevated levels of hemoglobin despite rearing in normoxia for generations (Boggs and Gross, 2025). Given that the transcriptome can change markedly when comparing captive-bred versus wild-caught individuals (Krishnan et al., 2020), an essential future direction for this research includes examination of the blood transcriptome from individuals drawn from the natural population.

## Materials and methods

### Animal husbandry and tissue collection

*Astyanax* cave and surface fish were reared in a satellite aquatic facility at the University of Cincinnati within a custom-designed reverse osmosis husbandry unit comprised of 5- and 10- gallon continuous flow tanks (Aquaneering, San Diego, CA). Animals were exposed to a 12:12 h light: dark cycle and fed a slurry of dry flake food (TetraMin Pro) and system water daily. Water in this system is processed through a series of filters including UV, 25-micron polypropylene felt, activated carbon, and dense particulate. Additionally, water conditions were adjusted using real-time dose monitoring of sodium bicarbonate and Instant Ocean sea salt to conductivity of 750 μS/cm (±50 μS/cm) and pH of 7.4 (±0.2). Water temperature was kept at 24°C (±2°C). Importantly, dissolved oxygen was not manipulated for this study meaning all fish were exposed to ample oxygen.

The surface fish, Pachón cavefish and Tinaja cavefish used in this study were derived from breeding adults originally provided to our lab by Dr. Richard Borowsky (New York University). Specifically, the pedigrees used included Asty-152 and Asty-155 (surface fish), Asty-163 and Asty-138 (Pachón cavefish), and Asty-19 (Tinaja cavefish). Surface fish are descended from wild-caught individuals from the Río Sabinas and Río Valles drainages near Ciudad Valles in San Luis Potosí, Mexico. All Chica cavefish were acquired from the commercial pet trade. We extracted whole blood from (n = 4) surface, Pachón, Tinaja, and Chica populations (total n = 16) via the caudal vein using 31G syringes (BD Ultra-Fine™, BD Biosciences, San Jose, CA). In order to limit any potential effects outside the scope of this study, two male and two female fish were used from each population, fish were post-breeding age, and whole blood extractions were completed between 12:00 p.m - 1:00 p.m. All procedures were conducted in accordance with University of Cincinnati IACUC (Protocol# 22-01-06-01).

### RNA isolation, sequencing, and read processing

Immediately following whole blood extraction, whole RNA was isolated using an RNeasy Universal Mini Kit (Qiagen, Germantown, MD) according to the manufacturer’s directions. All RNA samples were subjected to quantification using a Nanodrop Lite spectrophotometer (Thermo Fisher Scientific Inc., Waltham, MA). The purity of samples was estimated based on the A260/A280 ratio, and only RNA samples of ∼2.0 were submitted for sequencing. Owing to the technical requirements of RNA-sequencing, samples had to be pooled by population. To mitigate potential effects of sequencing error, each pool was sub-aliquoted into three technical replicates with each replicate (n = 12) containing the same volume of RNA. Pools were submitted to the DNA Core at Cincinnati Childrens’ Hospital and Medical Center. There, additional RNA QC was conducted, polyA stranded libraries were generated, QC was conducted on the libraries, and they were subject to sequencing using an Illumina HiSeq 2,500 sequencer. This resulted in twenty-million 125bp paired end reads per sample. Raw reads were assessed for quality and length using FastQC (Wingett and Andrews, 2018) (version 0.11.8) and adapters were trimmed using Trimmomatic (Bolger et al., 2014) (version 0.39).

### RNA sequencing

Analysis of gene expression was conducted by running a reference based analysis in CLC Genomics (Qiagen, Germantown, MD, version 12.0.1) using manufacturer recommended parameters. The latest *Astyanax* genome (AstMex3_surface, GCA_023375975.1) was used as the reference sequence. We used the latest annotation file for this reference from NCBI RefSeq (GCF_023375975.1, NCBI annotation release 103).

In order to increase efficiency of downstream transcriptome-wide analysis, we conducted a second RNA sequencing experiment in CLC Genomics using the “Astyanax-mexicanus-2.0” genome retrieved from Ensembl [GCA_000372685.2 (Warren et al., 2021)] as the reference and annotations from Ensembl release 106 were used to identify genes and determine expression. RNA-sequencing was validated using qPCR for five genes [see Boggs and Gross (2025)].

### Gene ontology enrichment and candidate gene nomination

To investigate transcriptome-wide patterns of gene expression, we conducted a Gene Ontology (GO) Statistical Overrepresentation Test. Each cave population was assessed independently against surface fish. Thus, we created seven gene lists, one list representing genes expressed higher in a cave population versus surface fish, one list representing genes expressed lower in a cave population versus surface, and a list containing all genes detectable in this assay [noise threshold surpassed with TPM value of at least 2 (Wagner et al., 2013)]. Each list representing a comparison between a cave and surface population contained genes detectable for at least one of the two populations and with a fold change of at least 2x (any gene with a TPM value of 0 was substituted with the lowest TPM value in the entire dataset - 0.00192,433 in Tinaja *fat1a*–so that a fold change value could be calculated). We used PANTHERdb (Mi et al., 2019; Thomas et al., 2022) (version 17.0) to conduct a statistical overrepresentation test. Because *Astyanax* GO terms are not available in PANTHER, IDs in each list were converted to orthologous *Danio rerio* IDs by using BioMart (Smedley et al., 2009). We successfully converted 6,882 of 8,550 (∼80%) IDs from our and used these as our reference (Aleksander et al., 2023) for the statistical overrepresentation test. We used a Fisher’s Exact text to calculate p-values which were corrected using false discovery rate to determine statistical significance. Each of three categories of GO terms were assessed: biological process, molecular function, and cellular component. Results from each cave-to-surface analysis were then compared to determine convergence/divergence between cave populations.

In addition, we investigated genes of potential interest that may have been missed in the GO analysis. Thus, we compiled four additional lists of genes: two lists representing genes of putative biological relevance that are either expressed higher or lower in Chica, Tinaja, and Pachón cavefish compared to surface fish as well as two lists expressed higher or lower in Tinaja and Pachón compared to surface. Expression data derived from Chica cavefish was omitted from these lists owing to the difference in expression of *hemoglobin* compared to Tinaja and Pachón. Genes were ranked according to putative biological relevance. Rank was determined by subtracting the fold change (cavefish expression value divided by surface fish expression value) of a gene from each cavefish expression value and summing the absolute values from each cave population. Genes that have not been characterized were removed and the remaining genes were filtered for relevance to hypoxia using literature searches.

## Data Availability

The datasets presented in this study can be found in online repositories. The names of the repository/repositories and accession number(s) can be found below: https://www.ncbi.nlm.nih.gov/, BioProject PRJNA1079358.

## References

[B1] Aleksander S. A. Balhoff J. Carbon S. Cherry J. M. Drabkin H. J. Ebert D. (2023). The gene ontology knowledgebase in 2023. Genetics 224, iyad031. 10.1093/genetics/iyad031 36866529 PMC10158837

[B3] Arsham A. M. Howell J. J. Simon M. C. (2003). A novel hypoxia-inducible factor-independent hypoxic response regulating mammalian target of rapamycin and its targets. J. Biol. Chem. 278, 29655–29660. 10.1074/jbc.M212770200 12777372

[B4] Basu B. Wang W. Ruppin R. Hannenhalli H. (2021). Predicting tissue-specific gene expression from whole blood transcriptome. Sci. Adv. 7, eabd6991. 10.1126/sciadv.abd6991 33811070 PMC11057699

[B5] Boggs T. E. Friedman J. S. Gross J. B. (2022). Alterations to cavefish red blood cells provide evidence of adaptation to reduced subterranean oxygen. Sci. Rep. 12, 3735. 10.1038/s41598-022-07619-0 35260642 PMC8904627

[B6] Boggs T. E. Gross J. B. (2025). Elevated blood hemoglobin in different cavefish populations evolves through diverse hemoglobin gene expression patterns. J. Exp. Zoology Part B Mol. Dev. Evol. 344, 175–181. 10.1002/jez.b.23289 PMC12046277 39930703

[B7] Bolger A. M. Lohse M. Usadel B. (2014). Trimmomatic: a flexible trimmer for illumina sequence data. Bioinformatics 30, 2114–2120. 10.1093/bioinformatics/btu170 24695404 PMC4103590

[B8] Brugarolas J. Lei K. Hurley R. L. Manning B. D. Reiling J. H. Hafen E. (2004). Regulation of mTOR function in response to hypoxia by REDD1 and the TSC1/TSC2 tumor suppressor complex. Genes and Dev. 18 (23), 2893–2904. 10.1101/gad.1256804 15545625 PMC534650

[B9] Bruick R. K. McKnight S. L. (2001). A conserved family of Prolyl-4-Hydroxylases that modify HIF. Science 294, 1337–1340. 10.1126/science.1066373 11598268

[B10] Chen N. Chen L. P. Zhang J. Chen C. Wei X. L. Gul Y. (2012). Molecular characterization and expression analysis of three hypoxia-inducible factor alpha subunits, HIF-1α/2α/3α of the hypoxia-sensitive freshwater species, Chinese sucker. Gene. 498, 81–90. 10.1016/j.gene.2011.12.058 22342256

[B11] Chen P. Chiu W. Hsu P. Lin S. Peng I. Wang C. (2020). Pathophysiological implications of hypoxia in human diseases. J. Biomed. Sci. 27, 63. 10.1186/s12929-020-00658-7 32389123 PMC7212687

[B12] Chun Y. Kim J. (2021). AMPK–mTOR signaling and cellular adaptations in hypoxia. Int. J. Mol. Sci. 22, 9765. 10.3390/ijms22189765 34575924 PMC8465282

[B13] Clees A. Stolp V. Häupl B. Fuhrmann D. C. Wempe F. Seibert M. (2022). Identification of the cysteine protease legumain as a potential chronic hypoxia-specific multiple myeloma target gene. Cells 11, 292. 10.3390/cells11020292 35053409 PMC8773999

[B14] Cohen I. Huang Y. Chen J. Benesty J. Benesty J. Chen J. (2009). “Pearson correlation coefficient,” in Noise reduction in speech processing, 1–4.

[B15] Diao X. Ye F. Zhang M. Ren X. Tian X. Lu J. (2022). Identification of oleoylethanolamide as an endogenous ligand for HIF-3α. Nat. Commun. 13 (1), 2529. 10.1038/s41467-022-30338-z 35534502 PMC9085743

[B16] Elliott W. R. (2018). The astyanax caves of Mexico: Cavefishes of Tamaulipas, San Luis Potosí, and Guerrero. Assoc. Mexican Cave Stud. 26, 1–325.

[B17] Epstein A. C. R. Gleadle J. M. McNeill L. A. Hewitson K. S. O'Rourke J. Mole D. R. (2001). *C. elegans* EGL-9 and mammalian homologs define a family of dioxygenases that regulate HIF by prolyl hydroxylation. Cell. 107, 43–54. 10.1016/s0092-8674(01)00507-4 11595184

[B18] Estrella N. L. Desjardins C. A. Nocco S. E. Clark A. L. Maksimenko Y. Naya F. J. (2015). MEF2 transcription factors regulate distinct gene programs in Mammalian skeletal muscle differentiation. J. Biol. Chem. 290, 1256–1268. 10.1074/jbc.M114.589838 25416778 PMC4294490

[B19] Evensen N. A. Li Y. Kuscu C. Liu J. Cathcart J. Banach A. (2015). Hypoxia promotes Colon cancer dissemination through up-regulation of cell migration-inducing protein (CEMIP). Oncotarget 6, 20723–20739. 10.18632/oncotarget.3978 26009875 PMC4653038

[B20] Fago A. Jensen F. B. (2015). Hypoxia tolerance, nitric oxide, and nitrite: lessons from extreme animals. Physiology 30, 116–126. 10.1152/physiol.00051.2014 25729057

[B21] Favaro E. Ramachandran A. McCormick R. Gee H. Blancher C. Crosby M. (2010). MicroRNA-210 regulates mitochondrial free radical response to hypoxia and krebs cycle in cancer cells by targeting iron sulfur cluster protein ISCU. Plos One 5, e10345. 10.1371/journal.pone.0010345 20436681 PMC2859946

[B22] Fingar D. C. Salama S. Tsou C. Harlow E. D. Blenis J. (2002). Mammalian cell size is controlled by mTOR and its downstream targets S6K1 and 4EBP1/eIF4E. Genes Dev. 16, 1472–1487. 10.1101/gad.995802 12080086 PMC186342

[B23] Foltyn M. Luger A. L. Lorenz N. I. Sauer B. Mittelbronn M. Harter P. N. (2019). The physiological mTOR complex 1 inhibitor DDIT4 mediates therapy resistance in glioblastoma. Br. J. Cancer 120 (5), 481–487. 10.1038/s41416-018-0368-3 30745581 PMC6461855

[B24] Fumey J. Hinaux H. Noirot C. Thermes C. Rétaux S. Casane D. (2018). Evidence for late Pleistocene origin of Astyanax mexicanus cavefish. BMC Evol. Biol. 18, 43. 10.1186/s12862-018-1156-7 29665771 PMC5905186

[B25] Garduño-Sánchez M. Hernández-Lozano J. Moran R. L. Miranda-Gamboa R. Gross J. B. Rohner N. (2023). Phylogeographic relationships and morphological evolution between cave and surface Astyanax mexicanus populations (de filippi 1853) (actinopterygii, characidae). Mol. Ecol. 10.1111/mec.17128 37712324

[B26] Goyal R. Longo L. D. (2014). Acclimatization to long-term hypoxia: gene expression in ovine carotid arteries. Physiol. Genomics 46, 725–734. 10.1152/physiolgenomics.00073.2014 25052263 PMC4187120

[B27] Herman A. Brandvain Y. Weagley J. Jeffery W. R. Keene A. C. Kono T. J. Y. (2018). The role of gene flow in rapid and repeated evolution of cave-related traits in Mexican tetra, Astyanax mexicanus. Mol. Ecol. 27, 4397–4416. 10.1111/mec.14877 30252986 PMC6261294

[B28] Hu Y. Zhang T. Chen J. Cheng W. Chen J. Zheng Z. (2020). Downregulation of hypoxia-inducible Factor-1α by RNA interference alleviates the development of collagen-induced arthritis in rats. Mol. Ther. - Nucleic Acids 19, 1330–1342. 10.1016/j.omtn.2020.01.014 32160704 PMC7038004

[B29] Ishihama S. Yoshida S. Yoshida T. Mori Y. Ouchi N. Eguchi S. (2021). LPL/AQP7/GPD2 promotes glycerol metabolism under hypoxia and prevents cardiac dysfunction during ischemia. FASEB J. 35, e22048. 10.1096/fj.202100882R 34807469

[B30] Ivan M. Kondo K. Yang H. Kim W. Valiando J. Ohh M. (2001). HIFalpha targeted for VHL-Mediated destruction by proline hydroxylation: implications for O2 sensing. Science 292, 464–468. 10.1126/science.1059817 11292862

[B31] Jaakkola P. Mole D. R. Tian Y. Wilson M. I. Gielbert J. Gaskell S. J. (2001). Targeting of HIF-α to the von Hippel-Lindau Ubiquitylation Complex by O2-Regulated Prolyl Hydroxylation. Science. 292, 468–472. 10.1126/science.1059796 11292861

[B32] Jung Y. Kim M. Soh H. Lee S. Kim J. Park S. (2014). TC1(C8orf4) regulates hematopoietic stem/progenitor cells and hematopoiesis. Plos One 9, e100311. 10.1371/journal.pone.0100311 24937306 PMC4061086

[B33] Khayami R. Hashemi S. R. Kerachian M. A. (2020). Role of aldo‐keto reductase family 1 member B1 (AKR1B1) in the cancer process and its therapeutic potential. J. Cell Mol. Med. 24, 8890–8902. 10.1111/jcmm.15581 32633024 PMC7417692

[B34] Kishimoto K. Yoshida S. Ibaragi S. Yoshioka N. Okui T. Hu G. (2012). Hypoxia-induced up-regulation of angiogenin, besides VEGF, is related to progression of oral cancer. Oral Oncol. 48, 1120–1127. 10.1016/j.oraloncology.2012.05.009 22694909

[B35] Krishnan J. Persons J. L. Peuß R. Hassan H. Kenzior A. Xiong S. (2020). Comparative transcriptome analysis of wild and lab populations of *Astyanax mexicanus* uncovers differential effects of environment and morphotype on gene expression. J. Exp. Zool. Mol. Dev. Evol. 334, 530–539. 10.1002/jez.b.22933 32017448

[B36] Krishnan J. Wang Y. Kenzior O. Hassan H. Olsen L. Tsuchiya D. (2022). Liver-derived cell lines from cavefish Astyanax mexicanus as an *in vitro* model for studying metabolic adaptation. Sci. Rep. 12, 10115. 10.1038/s41598-022-14507-0 35710938 PMC9203785

[B37] Li Z. Yang Y. Wu K. Li Y. Shi M. (2023). Myeloid leukemia factor 1: a “double-edged sword” in health and disease. Front. Oncol. 13, 1124978. 10.3389/fonc.2023.1124978 36814822 PMC9939472

[B38] Mandic M. Joyce W. Perry S. F. (2021). The evolutionary and physiological significance of the hif pathway in teleost fishes. J. Exp. Biol. 224, jeb231936. 10.1242/jeb.231936 34533194

[B39] Maxwell P. H. Wiesener M. S. Chang G. Clifford S. C. Vaux E. C. Cockman M. E. (1999). The tumour suppressor protein VHL targets hypoxia-inducible factors for oxygen-dependent proteolysis. Nature 399, 271–275. 10.1038/20459 10353251

[B40] Mi H. Muruganujan A. Huang X. Ebert D. Mills C. Guo X. (2019). Protocol update for large-scale genome and gene function analysis with the PANTHER classification system (v. 14.0). Nat. Protoc. 14, 703–721. 10.1038/s41596-019-0128-8 30804569 PMC6519457

[B41] Miranda-Gamboa R. Espinasa L. de los Angeles Verde-Ramírez M. Hernández-Lozano J. Lacaille J. L. Espinasa M. (2023). A new cave population of Astyanax mexicanus from Northern Sierra de El Abra, Tamaulipas, Mexico. Subterr. Biol. 45, 95–117. 10.3897/subtbiol.45.98434

[B42] Moran R. L. Richards E. J. Ornelas-García C. P. Gross J. B. Donny A. Wiese J. (2023). Selection-driven trait loss in independently evolved cavefish populations. Nat. Commun. 14, 2557. 10.1038/s41467-023-37909-8 37137902 PMC10156726

[B43] Murphy T. E. Rees B. B. (2024). Diverse responses of *hypoxia-inducible factor alpha* mRNA abundance in fish exposed to low oxygen: the importance of reporting methods. Front. Physiology 15, 1496226. 10.3389/fphys.2024.1496226 PMC11486919 39429981

[B44] Nikinmaa M. Rees B. B. (2005). Oxygen-dependent gene expression in fishes. Am. J. Physiology-Regulatory, Integr. Comp. Physiology 288, R1079–R1090. 10.1152/ajpregu.00626.2004 15821280

[B45] Pelster B. Egg M. (2018). Hypoxia-inducible transcription factors in fish: expression, function and interconnection with the circadian clock. J. Exp. Biol. 221, jeb163709. 10.1242/jeb.163709 29973414

[B46] Prchal M. D'Ambrosio J. Lagarde H. Lallias D. Patrice P. François Y. (2023). Genome-wide association study and genomic prediction of tolerance to acute hypoxia in rainbow trout. Aquaculture 565, 739068. 10.1016/j.aquaculture.2022.739068

[B47] Riddle M. R. Aspiras A. C. Damen F. Hutchinson J. N. Chinnapen D. J.-Tabin J. (2020). Genetic architecture underlying changes in carotenoid accumulation during the evolution of the blind Mexican cavefish, Astyanax mexicanus. J. Exp. Zool. Mol. Dev. Evol. 334, 405–422. 10.1002/jez.b.22954 PMC7708440 32488995

[B48] Rytkönen K. T. Renshaw G. M. Vainio P. P. Ashton K. J. Williams-Pritchard G. Leder E. H. (2012). Transcriptional responses to hypoxia are enhanced by recurrent hypoxia (Hypoxic preconditioning) in the epaulette shark. Physiol. genomics 44 (22), 1090–1097. 10.1152/physiolgenomics.00081.2012 22991209

[B49] San L. Liu B. Liu B. Zhu K. Guo L. Guo H. (2021). Genome-wide association study reveals multiple novel SNPs and putative candidate genes associated with low oxygen tolerance in golden pompano Trachinotus ovatus (linnaeus 1758). Aquaculture 544, 737098. 10.1016/j.aquaculture.2021.737098

[B50] Semenza G. L. Wang G. L. (1992). A Nuclear Factor Induced by Hypoxia *via de novo* Protein Synthesis Binds to the Human Erythropoietin Gene Enhancer at a Site Required for Transcriptional Activation. Mol. Cell Biol. 12, 5447–5454. 10.1128/mcb.12.12.5447 1448077 PMC360482

[B51] Sethumadhavan S. Silva M. Philbrook P. Nguyen T. Hatfield S. M. Ohta A. (2017). Hypoxia and hypoxia-inducible factor (HIF) downregulate antigen-presenting MHC class I molecules limiting tumor cell recognition by T cells. Plos One 12, e0187314. 10.1371/journal.pone.0187314 29155844 PMC5695785

[B52] Sheng J. Xu Z. (2016). Three decades of research on angiogenin: a review and perspective. Acta Biochim. Biophys. Sin. 48, 399–410. 10.1093/abbs/gmv131 26705141 PMC4888354

[B53] Smedley D. Haider S. Ballester B. Holland R. London D. Thorisson G. (2009). BioMart–biological queries made easy. BMC Genomics 10, 22–12. 10.1186/1471-2164-10-22 19144180 PMC2649164

[B54] Storz J. F. (2016). Gene duplication and evolutionary innovations in hemoglobin-oxygen transport. Physiology 31 (3), 223–232. 10.1152/physiol.00060.2015 27053736 PMC5005275

[B55] Storz J. F. (2018). Hemoglobin: insights into protein structure, function, and evolution. Oxford University Press.

[B56] Tammali R. Saxena A. Srivastava S. K. Ramana K. V. (2011). Aldose reductase inhibition prevents hypoxia-induced increase in hypoxia-inducible factor-1alpha (HIF-1alpha) and vascular endothelial growth factor (VEGF) by regulating 26 S proteasome-mediated protein degradation in human Colon cancer cells. J. Biol. Chem. 286, 24089–24100. 10.1074/jbc.M111.219733 21576240 PMC3129190

[B57] Thomas P. D. Ebert D. Muruganujan A. Mushayahama T. Albou L. Mi H. (2022). PANTHER: making genome‐scale phylogenetics accessible to all. Protein Sci. 31, 8–22. 10.1002/pro.4218 34717010 PMC8740835

[B58] van der Weele C. M. Jeffery W. R. (2022). Cavefish cope with environmental hypoxia by developing more erythrocytes and overexpression of hypoxia-inducible genes. eLife 11, e69109. 10.7554/eLife.69109 34984980 PMC8765751

[B59] Wagner G. P. Kin K. Lynch V. J. (2013). A model based criterion for gene expression calls using RNA-Seq data. Theory Biosci. 132, 159–164. 10.1007/s12064-013-0178-3 23615947

[B60] Warren W. C. Boggs T. E. Borowsky R. Carlson B. M. Ferrufino E. Gross J. B. (2021). A chromosome-level genome of Astyanax mexicanus surface fish for comparing population-specific genetic differences contributing to trait evolution. Nat. Commun. 12, 1447. 10.1038/s41467-021-21733-z 33664263 PMC7933363

[B61] Watson O. Novodvorsky P. Gray C. Rothman A. M. K. Lawrie A. Crossman D. C. (2013). Blood flow suppresses vascular notch signalling *via* dll4 and is required for angiogenesis in response to hypoxic signalling. Cardiovasc Res. 100, 252–261. 10.1093/cvr/cvt170 23812297 PMC3797625

[B62] Wijesooriya K. Jadaan S. A. Perera K. L. Kaur T. Ziemann M. (2022). Urgent need for consistent standards in functional enrichment analysis. PLOS Comput. Biol. 18, e1009935. 10.1371/journal.pcbi.1009935 35263338 PMC8936487

[B2] Wingett S. W. Andrews S.(2018). FastQ Screen: A tool for multi-genome mapping and quality control. F1000 Research 7, 1338. 30254741 10.12688/f1000research.15931.1 PMC6124377

[B63] Winteringham L. N. Kobelke S. Williams J. H. Ingley E. Klinken S. P. (2004). Myeloid leukemia factor 1 inhibits erythropoietin-induced differentiation, cell cycle exit and p27Kip1 accumulation. Oncogene 23, 5105–5109. 10.1038/sj.onc.1207661 15122318

[B64] Xiao W. (2015). The hypoxia signaling pathway and hypoxic adaptation in fishes. Sci. China Life Sci. 58, 148–155. 10.1007/s11427-015-4801-z 25595051

[B65] Xiaofei C. Yanqing L. Dongkai Z. Dong C. Feng Z. Weilin W. (2018). Identification of cathepsin B as a novel target of hypoxia-inducible factor-1-alpha in HepG2 cells. Biochem. Biophysical Res. Commun. 503 (2), 1057–1062. 10.1016/j.bbrc.2018.06.116 29935187

[B66] Yamasaki M. Nomura T. Sato F. Mimata H. (2007). Metallothionein is up-regulated under hypoxia and promotes the survival of human prostate cancer cells. Oncol. Rep. 18, 1145–1153. 10.3892/or.18.5.1145 17914565

[B67] Yang S. L. Wu C. Xiong Z. F. Fang X. (2015). Progress on hypoxia-inducible factor-3: its structure, gene regulation and biological function (review). Mol. Med. Rep. 12 (2), 2411–2416. 10.3892/mmr.2015.3689 25936862

[B68] Zhou C. H. Zhang X. P. Liu F. Wang W. (2015). Modeling the interplay between the HIF-1 and p53 pathways in hypoxia. Sci. Rep. 5 (1), 13834. 10.1038/srep13834 26346319 PMC4561886

